# Habituation to thaxtomin A increases resistance to common scab in ‘Russet Burbank’ potato

**DOI:** 10.1371/journal.pone.0253414

**Published:** 2021-06-16

**Authors:** Nathalie Beaudoin, Iauhenia Isayenka, Audrey Ducharme, Sophie Massie, André Gagnon, Richard Hogue, Carole Beaulieu, Dominique Michaud

**Affiliations:** 1 Département de biologie, Centre SÈVE, Université de Sherbrooke, Sherbrooke, QC, Canada; 2 Progest 2001, Sainte-Croix, QC, Canada; 3 Institut de recherche et de développement en agroenvironnement inc. (IRDA), Québec, QC, Canada; 4 Centre de recherche et d’innovation sur les végétaux, Université Laval, Québec, QC, Canada; National University of Kaohsiung, TAIWAN

## Abstract

Common scab is a potato disease characterized by the formation of scab-like lesions on the surface of potato tubers. The actinobacterium *Streptomyces scabiei* is the main causal agent of common scab. During infection, this bacterium synthesizes the phytotoxin thaxtomin A which is essential for the production of disease symptoms. While thaxtomin A can activate an atypical programmed cell death in plant cell suspensions, it is possible to gradually habituate plant cells to thaxtomin A to provide resistance to lethal phytotoxin concentrations. Potato ‘Russet Burbank’ calli were habituated to thaxtomin A to regenerate the somaclone RB9 that produced tubers more resistant to common scab than those obtained from the original cultivar. Compared to the Russet Burbank cultivar, somaclone RB9 generated up to 22% more marketable tubers with an infected tuber area below the 5% threshold. Enhanced resistance was maintained over at least two years of cultivation in the field. However, average size of tubers was significantly reduced in somaclone RB9 compared to the parent cultivar. Small RB9 tubers had a thicker phellem than Russet Burbank tubers, which may contribute to improving resistance to common scab. These results show that thaxtomin A-habituation in potato is efficient to produce somaclones with increased and durable resistance to common scab.

## Introduction

Common scab is a potato disease causing the formation of deep or superficial corky lesions on the surface of young developing tubers. This disease affects tuber market values as infected tubers with more than 5% of scab lesions on their surface are generally rejected by the industry. Several environmental conditions can promote the occurrence of common scab, making its management very complex. Approaches to control common scab include maintaining high soil moisture, changes in soil pH, crop rotation, soil fumigation and biological control, but these practices are not always successful (rev. in [1]). Hence, cultivation of potato cultivars that are more resistant to the disease comes out as a good approach to decrease common scab incidence. However, the lack of molecular markers linked to common scab resistance has impeded the development of resistant cultivars. Despite years of research, there is still little known about the mechanisms that protect potato tubers from common scab.

The main causal agent of common scab is *Streptomyces scabiei* (syn. *S*. *scabies*), an actinobacterium that synthesizes the phytotoxin thaxtomin A (TA), which is essential during tuber infection and for the development of scab lesions [2–4]. Intensity of disease symptoms has been correlated with the level of TA produced by *S*. *scabiei* [5]. Moreover, a *S*. *scabiei* mutant unable to synthesize TA did not cause scab symptoms [6, 7]. Based on its central role during *S*. *scabiei* infection, TA has been used to screen for potato cultivars resistant to common scab [8, 9]. However, weak sensitivity to TA in tubers was not always correlated with resistance to the disease [10]. Very high concentrations (4.57 and 6.86 μM) of TA have also been used to select potato cells that were resistant to TA [11, 12]. This has led to the production of a wide collection of somaclonal variants, including some mutants that were shown to be more resistant to TA and common scab, suggesting that increasing resistance to TA in potato may provide enhanced resistance to common scab.

At the cellular level, TA has been shown to inhibit cellulose biosynthesis [13]. TA treatment of plant seedlings induces shoot and root swelling, cell hypertrophy, changes in Ca^2+^ and H^+^ fluxes, accumulation of antimicrobial compound scopoletin and lignin deposition [13–17]. When added to Arabidopsis cell suspensions, TA induces an atypical programmed cell death (PCD) which does not involve the expression of typical defense related responses or the production of reactive oxygen species (ROS) [18, 19]. In this case, these effects were attributed to TA’s ability to inhibit cellulose synthesis, as the inhibitor of cellulose synthesis isoxaben also induced a similar PCD [18, 20]. More recently, TA was shown to activate the plant immunity-related Enhanced Disease Susceptibility 1 (EDS1)-dependent and Phytoalexin Deficient 4 (PAD4)-dependent pathway independently of its effect on cellulose biosynthesis [21]. Overall, these results suggest that TA can affect different cellular processes.

Although addition of TA to plant cells can be lethal [18, 19], there is experimental evidence that changes in cellular auxin or redox homeostasis can alleviate the effect of TA. Hence, pretreatments of cell suspensions with hydrogen peroxide or auxins hindered the induction of PCD by TA [22, 23]. Similarly, increased resistance to TA in the Arabidopsis mutant *txr1-1*, which is mutated in the *AtPAM16* gene coding for a mitochondrial negative regulator of ROS production, was associated with elevated ROS production [13, 24]. However, it is not known how ROS may inhibit TA toxicity.

It is also possible to progressively adapt or “habituate” plant cells to make them resistant to TA by adding incremental concentrations of TA at each subculture round [25]. Resistance to TA in TA-habituated poplar cells was maintained over several years and was associated with an important reprogramming of gene expression including genes involved in cell wall and lignin biosynthesis [25].

Since TA is a central factor in the development of common scab symptoms, we hypothesized that TA-habituation may be used to produce potato somaclones with increased resistance to the disease. We developed a new method which involved a progressive TA adaptation of ‘Russet Burbank’ potato calli that were first grown on low levels of TA (0.2 μM) up to growth inhibitory TA concentrations (0.6 μM). Somaclones were regenerated from TA-habituated calli and tested for resistance to common scab. The TA-habituated somaclone referred to as RB9 showed enhanced resistance to common scab in growth room infection assays and in field trials in comparison to the original Russet Burbank cultivar. Further characterization of RB9 should provide key information on the mechanisms underlying enhanced resistance to common scab.

## Materials and methods

### Plant material

Potato (*Solanum tuberosum* L.) ‘Russet Burbank’ tissue-cultured virus-free plantlets were graciously obtained from Les Semences Élite du Québec (Pointe-aux-Outardes, QC, Canada) and Centre de recherche Les Buissons (Pointe-aux-Outardes, QC, Canada). All chemicals used for this study were from Sigma unless indicated otherwise. Plants were maintained *in vitro* in propagation medium containing Murashige and Skoog (MS) medium supplemented with 3% (w/v) sucrose and 0.7% (w/v) bactoagar (BD Difco), pH 5.7 and propagated every 5 to 8 weeks using stem cuttings with 1 or 2 nodes. Plants were cultivated either in Magenta vessel GA-7 or test tubes in a Sanyo MLR-350 chamber with a 16 h light photoperiod (75 μmol m^-2^ s^-1^) at 21°C.

### Thaxtomin A purification

Thaxtomin A (TA) was purified from *Streptomyces scabiei* EF-35 [26] cultivated in oat bran broth as described before [6]. Purified TA was quantified by HPLC using a Varian LC5500 liquid chromatograph equipped with a Water’s C18 column [18]. Thaxtomin A was diluted in methanol at a final concentration of 0.01 M.

### Calli production and habituation to thaxtomin A

**Fig 1:**
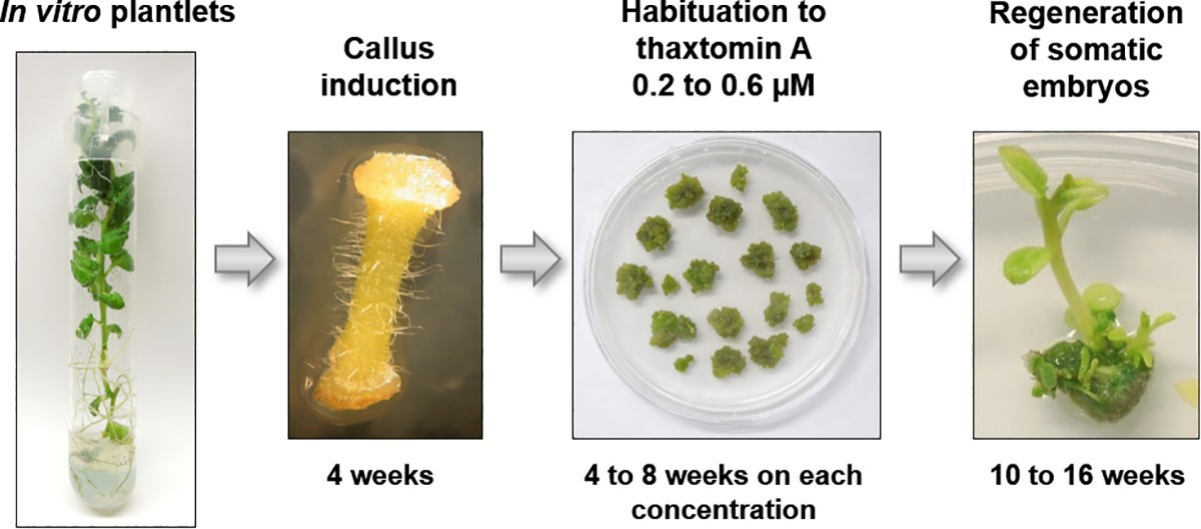
Thaxtomin A (TA)-habituation of potato calli and regeneration of somatic embryos. Calli produced from *in vitro* grown plantlets were cultivated on medium containing 0.2 μM TA for 4 weeks. Calli were repetitively transferred every 4 to 8 weeks to medium containing increased concentrations of TA each time. At each transfer, part of the TA-habituated calli was transferred to regeneration medium. See Materials and methods for further details.

Stem internodes (1 cm-long) were cut from *in vitro* grown potato plantlets and cultivated in the dark at 21°C on callus-inducing medium (CIM) containing MS medium, 3% (w/v) sucrose, 0.9 μM 2,4-dichlorophenoxyacetic acid (2,4-D), 10 μM benzylaminopurine (BA), 0.7% (w/v) bactoagar (BD Difco), pH 5.7 [27]. Calli formed after 4 weeks were excised and transferred to CIM supplemented with 0.2 μM TA. The same volume of methanol was added to TA-free CIM used for control calli. Every 4 to 8 weeks, calli were transferred on fresh CIM with TA concentration increased by 0.1 μM. This was repeated several times up to a concentration of 0.6 μM for a total duration of approximately 25 weeks (Fig 1).

### Regeneration of somatic embryos

Every time calli were transferred to a new TA concentration, some of the TA-habituated calli were transferred to a regeneration medium containing MS supplemented with 3% (w/v) sucrose, 10 μM BA, 22.8 μM zeatin and 0.7% (w/v) bactoagar (BD Difco), pH 5.7 [27]. Regenerating calli were incubated under a 16 h light photoperiod at 21°C. Somatic embryos were excised, labeled (RB1, RB2, etc.) and cultivated on propagation MS medium supplemented with 3% (w/v) sucrose and 0.7% (w/v) bactoagar. Only one somatic embryo was selected per callus to ensure that each regenerated TA-habituated somaclone was unique. TA-habituated somaclones were propagated *in vitro* as described above.

### Infection in pots

The bacterial inoculum was prepared with protocols modified from [26, 28]. *Streptomyces scabiei* EF-35 were cultivated at 30°C in liquid Yeast Malt Extract medium (YME) containing 4 g L^-1^ glucose, 4 g L^-1^ yeast extract (Fisher Scientific) and 10 g L^-1^ malt extract (BD Difco) for 5 to 8 days, at 250 rpm. Bacteria were plated on solid YME medium supplemented with 1.5% (w/v) agar and 1 g L^-1^ CaCO_3_ and incubated for 7 days at 30°C. Say solution containing 20 g L^-1^ glucose, 1.2 g L^-1^ asparagine, 0.6 g L^-1^ K_2_HPO_4_ and 10 g L^-1^ yeast extract (DF Difco) was mixed with 300 mL of autoclaved vermiculite in Magenta GA-7 boxes. Each Magenta box was inoculated with one plug (1 cm^2^) cut from solid YME covered with *S*. *scabiei* mycelium and incubated in the dark for 4 weeks at 30°C. Bacteria in boxes were mixed every week. The bacterial inoculum for plant infection was prepared by mixing thoroughly together all *S*. *scabiei* cultures grown in vermiculite.

For infection trials, Magenta vessel GA-7 boxes containing 3- to 4-week-old potato plantlets were transferred with the lid ajar to a Conviron growth chamber for 3 to 4 days at 21°C. Plants were then transferred to pots containing autoclaved sand and vermiculite (2:1) without bacteria (control) or thoroughly mixed with the bacteria inoculum (1:20). The plants were grown in a Conviron growth chamber at a 16 h light photoperiod and 21°C/16°C day/night for the first month and subsequently transferred to a 12 h light photoperiod to induce tuberization. Watering was restricted to 50 mL twice a week for the first 20 days, and slowly increased up to 200 mL after 50 days of growth. Plants were fertilized weekly with NPK (20-20-20). Tubers were harvested after 90 to 110 days and analysed for common scab symptoms.

### Common scab severity index (CSSI)

Common scab severity score for each tuber was evaluated using a scale of 0 to 6 relative to the percentage of the tuber surface covered by scabby lesions, with 0 = no scab; 0.5 = less than 1%; 1 = 1 to 5%; 2 = 6 to 10%; 3 = 11 to 25%; 4 = 26 to 50%; 5 = 51 to 75%; and 6 = 76 to 100%. The common scab severity index (CSSI) was calculated as the total of common scab severity scores divided by the total number of tubers for a given somaclone or original cultivar.

### Thaxtomin A sensitivity test

The intensity of tissue browning in response to TA was evaluated qualitatively to determine tubers sensitivity to TA. Mature tubers were washed with water, sterilized in 15% (w/v) bleach diluted in water for 20 min and dried under a laminar flow hood. Transverse slices were cut from the middle portion of the tuber and put on wet sterile filter paper in a petri dish. Sterile filter paper discs (7 mm diameter) soaked in different concentrations of TA (0.5 to 2.0 μM) diluted in methanol or methanol as a control were dried under the hood before being placed in the center of each slice with a drop of sterile water. Potato slices were incubated in the dark for 6 days. At least 6 different tubers from Russet Burbank (RBP) and somaclone RB9 were assayed with similar results.

### Field trials

For the first field trial in 2012, *in vitro* grown potato plantlets were transferred to pots containing soil, sand and vermiculite (2:1:1) and cultivated in a growth room for one week before being transplanted to cultivation plots in Saint-Thomas-de-Joliette, QC, Canada. These cultivation plots had a high prevalence of common scab in previous years. For each of RBP and RB9, plants were put in groups of 6 plants separated by 30 cm and arranged in 5 plots disposed alternately on rows separated by 92 cm. After harvest, each tuber was scored for common scab symptoms.

For the next field trials, potato tubers from RBP and RB9 were produced in pots in a greenhouse the previous summer using *in vitro* plantlets. Tubers were planted in fields which had a high incidence of common scab in the previous years at two different locations, i.e., Saint-Ambroise, QC and Sainte-Croix, QC, Canada. Field trials 1 (Saint-Ambroise, 2015), 2 (Sainte-Croix, 2015) and 3 (Sainte-Croix, 2016) included 4 plots for each of RBP and RB9, and field trial 4 (Saint-Ambroise, 2016) included 8 plots for each of RBP and RB9. Plants were separated by 30 cm and plots were randomly distributed on rows separated by 92 cm. After harvest, each tuber was weighed and scored for common scab symptoms.

Harvested tubers were graded with a mechanical sorter according to their size. Tubers normally rejected by growers (green, malformed, cracked, rotten) were also weighed. Each tuber was weighed and scored for common scab symptoms as described above. Ten tubers of average size were randomly selected from each cultivation plot and evaluated for black scurf using scores from the Canadian Food Inspection Agency (CFIA) with 0 = 0% infected surface area; 1 = 1%; 2 = 5%; 3 = 10% and 4 >15%. These tubers were then cut to evaluate internal defects, such as hollow heart, brown heart, and vascular discoloration. Specific gravity was also determined for RBP and RB9 tubers.

### Microscopical examination of periderm

Tubers harvested from plants grown in Conviron growth chamber were used to evaluate periderm organization and phellem thickness. Tubers were grouped according to weight and size with size 1 = 0.3–0.8 g (0.8–1.2 cm); size 2 = 1.0–1.7 g (1.3–1.7 cm); size 3 = 2.7–4.4 g (2.2–2.6 cm); and size 4 = 5.3–8.6 g (2.7–3.2 cm). Tubers were cut in 3 slices taken from the distal (stolon), middle and proximal (apex) regions of the tuber. Cross sections of the periderm were cut on 2 sides of each slice with a razor blade. The phellem portion of the periderm, which contains autofluorescent suberin, was visualized using a Zeiss AxioImager Z1 Fluorescent microscope (excitation 365 nm, emission 445/50 emission 461 nm; DAPI). Suberized cell layers were counted on photographs taken from different regions of the tuber. The average number of cell layers was calculated from 4 tubers.

### Statistical analysis

Statistical analyses were performed using GraphPad Prism 9.0. Statistical differences (*P*<0.05) for infected and marketable tubers were estimated by two-tailed Fisher’s exact test. Statistical differences (*P*<0.05) for CSSI, tuber size and phellem cell layers were estimated by Welch’s *t*-test (*P*<0.05). Finally, statistical differences (*P*<0.05) for yield and physiological characters were estimated by multiple *t*-tests with correction for multiple comparisons using the Holm-Sidak method. Datasets are available in S1 Table.

## Results

### Habituation to TA

Calli produced from *in vitro* grown potato ‘Russet Burbank’ (RBP) plantlets [27] were transferred to CIM supplemented with an initial concentration of 0.2 μM thaxtomin A (TA) (Fig 1), a concentration at which calli growth was similar in TA and control medium. After 4 weeks, calli were transferred to CIM with a TA concentration increased by 0.1 μM up to 0.3 μM TA. Calli growth on TA was slowed down compared to calli grown on CIM without TA. Since early transfer to higher levels of TA induced calli death, transfers to higher TA concentration were delayed by 5 to 8 weeks depending on the TA concentration. At each transfer, some calli were also transferred to regeneration medium for somatic embryo production [27]. Overall, seven somatic embryos produced viable plants during this process, including 5 which were habituated up to 0.4 μM TA and 2 up to 0.5 μM TA (Table 1).

**Table 1:** Somaclones regenerated from Thaxtomin A (TA)-habituated calli.

Viable somaclones regenerated from TA-habituated calli	Maximum TA concentration (μM) used for habituation	No. of weeks on TA-habituation medium	No. of weeks on regeneration medium
RB3, RB4, RB5, RB6, RB7	0.4	25	10 to 13
RB9, RB13	0.5	25	12 to 16

### Resistance to common scab and thaxtomin A

Three TA-habituated somaclones RB5, RB6 and RB9 were randomly selected to evaluate their resistance to common scab compared to that of the original cultivar RBP in a growth chamber assay. *In vitro*-cultured plantlets from somaclones and from RBP were cultivated in pots containing a mixture of sand and vermiculite inoculated or not with *S*. *scabiei* in a Conviron growth chamber, as described in Materials and methods. Potato tubers were harvested and evaluated for common scab symptoms. Control tubers grown in the absence of *S*. *scabiei* had no common scab symptoms. As shown in Table 2 (Trial 1), while there was no significant difference in the proportion of non-infected tubers, this proportion tended to be lower in RB9 (57.1%) than in RBP (70.0%), but higher in RB5 (83.8%) and RB6 (76.5%). The common scab severity index (CSSI) was significantly reduced in RB9 (0.33) compared to RBP (0.56) while it was significantly higher in RB5 (1.01) and RB6 (0.96). Based on these results, resistance to common scab was further tested for RB9 in two additional infection assays (Table 2; Trials 2 and 3). In both tests, less RB9 tubers were infected by common scab than RBP tubers. This reduction was significant in trial 3, with 65.7% infected tubers in RB9 compared to 90.5% in RBP. Moreover, the CSSI was decreased from 2.98 in RBP to 2.21 in RB9 in Trial 2, and from 2.12 in RBP down to 1.46 in RB9 in Trial 3. Overall, the proportion of tubers that had 5% or less surface covered by scab lesions, which are generally considered as marketable, was not significantly different in RB9 compared to RBP. Still, the proportion of marketable tubers was 7 to 18% higher in RB9 tubers than RBP tubers.

**Table 2:** Common scab occurrence in tubers from original ‘Russet Burbank’ (RBP) and TA-habituated somaclones cultivated in growth chamber.

TA-habituated somaclones	No. of tubers^x^	No. of infected tubers^y^	Common scab severity index ± SEM^z^	No. of tubers with ≤ 5% scabby surface^y^
**Trial 1 (2011)**				
RBP	40	28 (70.0%) a	0.56 ± 0.09 a	37 (92.5%) a
RB5	37	31 (83.8%) a	1.01 ± 0.16 b	28 (75.7%) a
RB6	34	26 (76.5%) a	0.96 ± 0.16 b	27 (79.4%) a
RB9	35	20 (57.1%) a	0.33 ± 0.05 c	35 (100.0%) a
**Trial 2 (2012)**				
RBP	55	49 (89.1%) a	2.98 ± 0.25 a	18 (32.7%) a
RB9	60	45 (75.0%) a	2.21 ± 0.26 b	26 (43.3%) a
**Trial 3 (2012)**				
RBP	42	38 (90.5%) a	2.12 ± 0.25 a	20 (47.6%) a
RB9	35	23 (65.7%) b	1.46 ± 0.29 a	23 (65.7%) a

^x^ Number of plants per somaclone: trial 1 = 4, trial 2 = 6, trial 3 = 12.

^y^ For each trial, proportions followed by a different letter were significantly different (*P* = 0.0108) according to two-tailed Fisher’s exact test.

^z^ For each trial, means followed by a different letter were significantly different (*P*<0.05) according to Welch’s *t*-test.

**Fig 2:**
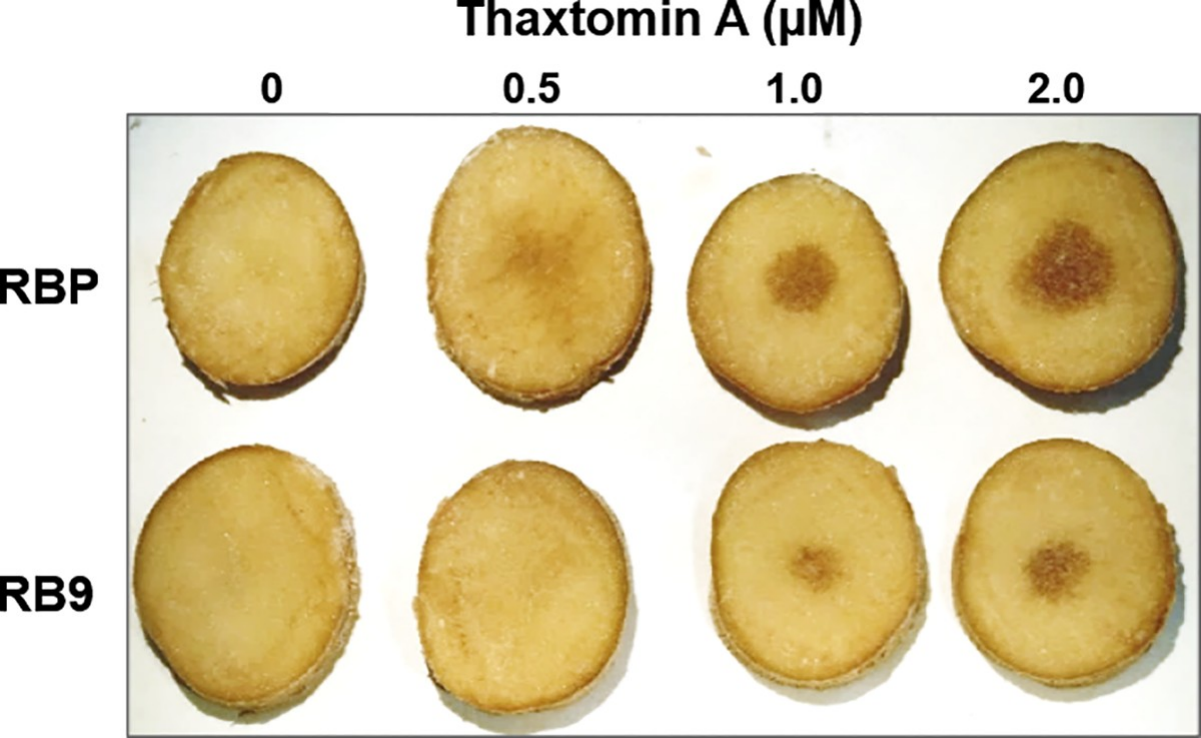
Decreased sensitivity to Thaxtomin A (TA) in TA-habituated somaclone RB9. Paper disks soaked in 0.5 to 2.0 μM TA or methanol (control) were laid on top of tuber slices of parent cultivar Russet Burbank (RBP) and somaclone RB9 tubers. Slices were incubated in the dark for 6 days. Paper disks were removed to take photos.

TA-habituated calli were more resistant to TA as they were able to proliferate on lethal TA concentrations. Hence, somaclones regenerated from TA-habituated calli may also be more resistant to TA. RB9 sensitivity to TA was tested in tubers. Thaxtomin A can induce browning in tubers correlated with toxin concentration or tuber sensitivity [3, 4, 29]. Under our conditions, RB9 tuber slices exposed to TA developed a less intense brown coloration than RBP tuber slices treated with a similar TA concentration (Fig 2), indicating that RB9 tubers were less sensitive to TA than RBP tubers.

### Field test using *in vitro* grown potato plantlets

Plants recovered from *in vitro* plantlets were cultivated in a field that showed high intensity of common scab in the previous years. The total number of tubers produced from RB9 plants was much smaller than the total number of tubers obtained from plants of the original cultivar RBP, with 318 tubers compared to 453 tubers (Table 3). However, due to the fragility of plants derived from *in vitro* grown material, it was not possible at this stage to conclude that the RB9 somaclone produces less tubers than the original cultivar. On the other hand, the incidence of common scab was significantly reduced in tubers from RB9 compared to RBP, with 75.8% infected tubers in RB9 compared to 86.7% in RBP. Moreover, the CSSI was reduced to 1.19 in RB9 compared to 1.99 in RBP, which also led to a significant 22% increase in the proportion of RB9 tubers which had 5% or less infected surface area. These results confirmed that the TA-habituated somaclone RB9 was more resistant to common scab than the original cultivar RBP.

**Table 3:** Field evaluation (2012) of common scab infection incidence and severity for parent Russet Burbank (RBP) and TA-habituated somaclone RB9.

Somaclone	No. of tubers^x^	No. of infected tubers^y^	Common scab severity index ± SEM^z^	No. of tubers with ≤ 5% scabby surface^y^
RBP	453	393 (86.7%) a	1.99 ± 0.08 a	227 (50.1%) a
RB9	318	241 (75.8%) b	1.19 ± 0.07 b	228 (71.7%) b

Plants were produced from TA-habituated *in vitro* grown plantlets.

^x^ Number of plants = 30 distributed in 5 plots per somaclone.

^y^ Proportions followed by a different letter within each column were significantly different (*P*≤0.0001) according to two-tailed Fisher’s exact test.

^z^ Means followed by a different letter were significantly different (*P*<0.0001) according to Welch’s *t*-test.

### Field trials with seed tubers

Resistance to common scab was evaluated in the field using tubers previously produced in a greenhouse. Fields for the trial were selected based on high incidence of common scab observed in previous years. However, the first field evaluation done in 2015 in Saint-Ambroise (Table 4, Field trial 1) showed very low scab infection. Nevertheless, harvested tubers were analysed for yield and physiological defects, as discussed below (Tables 5 and 6).

**Table 4:** Field evaluation of weight, common scab incidence and severity of tubers for parent cultivar Russet Burbank (RBP) and TA-habituated somaclone RB9.

Somaclone^v^^,^^w^	Total no. of tubers	Average tuber weight (g) ± SEM^x^	No. of infected tubers^y^^,^^z^	Common scab severity index ± SEM^x^	No. of tubers with ≤ 5% scabby surface^y^^,^^z^
**Field trial 1**					
RBP	90	104.0 ± 6.3 a	N/A	<0.03	N/A
RB9	135	69.0 ± 2.7 b	N/A	<0.03	N/A
**Field trial 2**					
RBP	192	98.4 ± 5.8 a	138 (71.9%) d	0.72 ± 0.07 a	170 (88.5%) d
RB9	192	62.5 ± 3.2 b	131 (68.2%) d	0.43 ± 0.03 b	187 (97.4%) e
**Field trial 3**					
RBP	186	134.8 ± 2.9 a	121 (65.0%) d	0.36 ± 0.02 a	186 (100.0%) d
RB9	189	94.4 ± 2.0 b	109 (57.7%) d	0.34 ± 0.02 a	187 (98.9%) d
**Field trial 4**					
RBP	879	67.8 ± 1.3 a	837 (95.2%) d	1.11 ± 0.02 a	738 (83.9%) d
RB9	1121	34.0 ± 0.6 b	1013 (90.4%) e	0.89 ± 0.02 c	1018 (90.8) e

^v^ Field trials 1 and 2 were performed in 2015; field trials 3 and 4 were performed in 2016.

^w^ Field trials 1 to 3: 4 plots; field trial 4: 8 plots.

^x^ Means followed by a different letter within a trial were significantly different at *P* = 0.0003 (b) and *P*<0.0001 (c) using Welch’s *t*-test.

^y^ N/A = Not available.

^z^ Proportions followed by a different letter within each column were significantly different (*P*≤0.0001) using two-tailed Fisher’s exact test.

**Table 5:** Yield of tubers as a function of tuber size for parent cultivar Russet Burbank (RBP) and somaclone RB9 grown in the field.

Somaclone^x^^,^^y^	Total yield	Yield (Mg ha^-1^)^z^ according to tuber size (mm)				
	(Mg ha^-1^)^z^	< 38	38 to 57	57 to 70	70 to 114	Rejected
**Field trial 1**						
RBP	35.63 a	2.42 a	5.20 a	24.84 a	3.18 a	0.00
RB9	28.34 a	8.36 b	9.36 b	10.61 b	0.00 b	0.00
**Field trial 3**						
RBP	70.17 a	6.89 a	9.94 a	50.24 a	1.02 a	2.08 a
RB9	42.10 b	10.89 a	11.86 a	19.06 b	0.00 a	0.29 a
**Field trial 4**						
RBP	22.58 a	7.01 a	7.30 a	7.65 a	0.00 a	0.63 a
RB9	13.25 b	9.89 a	3.11 a	0.19 b	0.00 a	0.06 a

^x^ These parameters were not evaluated in field trial 2.

^y^ Field trial 1 was performed in 2015; field trials 3 and 4 were performed in 2016.

^z^ Yields followed by different letters were statistically different (*P*<0.05) according to multiple *t*-tests with correction for multiple comparisons using the Holm-Sidak method.

**Table 6:** Physiological characteristics of tubers from Russet Burbank Parent (RBP) and somaclone RB9 grown in the field.

Somaclone^x^	Hollow heart (%)^y^	Brown heart (%)^y^	Vascular discoloration(%)^y^	Black scurf severity index^y^	Specific gravity^y^^,^ ^z^
**Field trial 1**					
RBP	22.5 a	0.0 a	7.5 a	0.30 a	N/A
RB9	0.0 a	0.0 a	5.0 a	0.10 a	N/A
**Field trial 3**					
RBP	0.0 a	5.0 a	42.5 a	1.70 a	1.089 a
RB9	0.0 a	0.0 a	45.0 a	0.50 a	1.084 a
**Field trial 4**					
RBP	0.0 a	5.0 a	2.5 a	1.48 a	1.092 a
RB9	0.0 a	1.3 a	6.3 a	1.83 a	1.088 a

^x^ These parameters were not evaluated in field trial 2.

^y^ Values are the means of 4 repeats (4 plots) with 10 tubers each. Means followed by the same letter were not statistically different (*P*<0.05) according to multiple *t*-tests with correction for multiple comparisons using the Holm-Sidak method.

^z^ N/A = not available.

The incidence of common scab infection was moderate in field trial 2 performed at Sainte-Croix in 2015 (Table 4). RB9 tubers had a CSSI of 0.43 that was significantly lower than that of 0.72 for RBP tubers. The proportion of tubers that had an infected surface area of 5% or less was significantly higher in RB9 (97%) compared to that from RBP (88%). Field trial 3 at Sainte-Croix in 2016 showed a weak level of infection. Common scab severity was similar in both RBP and RB9 tubers, with a CSSI of 0.36 and 0.34 in RBP and RB9, respectively. Field trial 4 at Saint-Ambroise in 2016 showed the highest level of common scab infection among all field trials. The proportion of infected RB9 tubers (90%) was reduced compared to that of RBP tubers (95%). In this trial, the CSSI was decreased to 0.89 in RB9 compared to 1.11 for RBP. Moreover, there was a significant increase in the proportion of marketable RB9 tubers that was close to 91% compared to 84% for the RBP tubers. Overall, these results indicate that RB9 tubers were significantly more resistant to common scab than RBP tubers in common scab infested fields, even several years after TA-habituation. However, no difference could be seen when the disease incidence was low, as observed in the field trial 3.

### Evaluation of yield and physiological parameters

Tubers harvested from field trials with seed tubers were weighed and sorted according to their size. As shown in Table 4, the average weight of RB9 tubers was significantly reduced in all field trials compared to that of RBP tubers. This also translated into a reduction of total yield for the RB9 somaclone in field trials 3 and 4 (Table 5). In particular, tubers smaller than 38 cm were prevalent in RB9 compared to RBP while tubers with size from 57 to 70 cm were significantly more abundant in RBP than in RB9. However, no significant differences were observed between RB9 and RBP tubers regarding the occurrence of physiological defects such as hollow heart, brown heart or vascular discoloration (Table 6). Moreover, RB9 tubers presented a similar specific gravity and were as sensitive as RBP tubers to black scurf (Table 6).

### Periderm analysis

**Fig 3:**
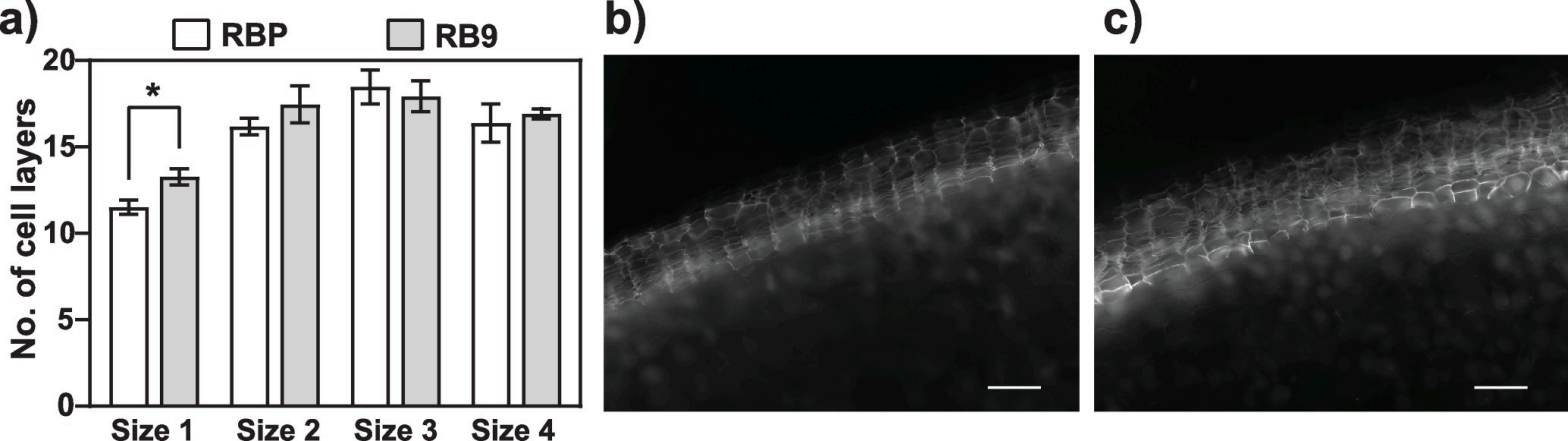
Periderm reinforcement in tubers from TA-habituated somaclone RB9. a) Number of phellem cell layers from parent cultivar Russet Burbank tubers (RBP; white bars) compared to RB9 tubers (grey bars) according to tuber size where size 1 = 0.8 to 1.2 cm; size 2 = 1.3 to 1.7 cm; size 3 = 2.2 to 2.6 cm; and size 4 = 2.7–3.2 cm. Values represent the average number of cell layers calculated from 4 different tubers ± SEM. Means labelled by * were significantly different (*P* = 0.031) using Welch’s t-test. b) and c) Micrographs of autofluorescent phellem cells in tubers from RBP (b) and RB9 (c). Bar = 100 μm.

Phellem cells, which form the outer layer of the tuber periderm, can be visualized by fluorescent microscopy due to the presence of autofluorescent suberin in their cell walls. Phellem cell layers in mature tubers of comparable size were visualised by fluorescent microscopy in RBP and RB9 tubers (Fig 3). The average number of cell layers was significantly increased in the smaller tubers of RB9, with an average of 13.3 cell layers compared to 11.5 in RBP tubers (Fig 3A). Moreover, phellem cells in RB9 appeared disorganized, with irregular shapes and sizes compared to RBP phellem cells which showed regular and generally rectangular shapes (Fig 3B and 3C). These observations suggest that TA-habituation and increased resistance to common scab in RB9 tubers was associated with modifications in the organization and thickness of the phellem cell layer.

## Discussion

One of the most efficient and environment friendly approaches to reduce common scab incidence is by cultivating potato cultivars that are more resistant to the disease. In this study, we developed a technique which led to the production of a potato somaclone that is more resistant to common scab than the original cultivar. The somaclone RB9 was regenerated from thaxtomin A (TA)-habituated Russet Burbank calli (Fig 1). Thaxtomin A is the main pathogenicity factor of the actinobacteria *S*. *scabiei* that causes common scab. This phytotoxin plays a central role in the development of common scab symptoms. Increasing resistance to TA in potato tubers stands out as a promising approach to enhance resistance to common scab. The mode of action of TA in the development of scab lesions remains uncertain. TA is an inhibitor of cellulose biosynthesis that was proposed to play a role in weakening the cell wall to allow *S*. *scabiei* to penetrate into tuber tissues [4, 30]. However, several studies indicate that TA affects different cellular mechanisms that are not intuitively linked with *S*. *scabiei* infection [13–18, 21].

The TA-habituation procedure used in this study involved growth of Russet Burbank calli on medium with concentrations of TA gradually increased up to 0.6 μM, a concentration at which regular calli stopped growing and eventually died (Fig 1). Somaclones were regenerated from calli habituated to 0.4 and 0.5 μM TA (Table 1), indicating that TA-habituated calli could efficiently regenerate into somatic embryos to form plantlets that were phenotypically similar to those of the parent cultivar Russet Burbank (RBP). This method differs from the cell selection technique described previously where very high and lethal concentrations (4.57 and 6.86 μM) of thaxtomin A were used to “select” cells that remained alive in the presence of TA [11, 12]. In this technique, only cells that survived to high concentrations of TA could be regenerated into plantlets, while TA-habituation involved a progressive process where cells slowly adapted to TA through several cell division cycles. Our results suggest that TA-habituation somehow reprogrammed cells to become more resistant to TA. Long-term TA-habituation in poplar cells stably resistant to TA was associated with an important reprogramming of gene expression, with enhanced expression of genes involved in cell wall and lignin biosynthesis [25]. These cells had a modified cell wall with increased pectin content that could compensate for reduced cellulose synthesis [25]. Although TA-habituation in poplar cells was performed over a full year compared to 25 weeks for potato calli, TA-habituation in potato calli may also induce some reprogramming of gene expression to establish resistance to TA. For instance, changes in gene expression have been reported in the common scab resistant mutant A380 that was obtained from cell selection on high TA concentrations [11, 31]. Transcriptional analyses will now be welcome to pinpoint the changes that occurred during TA-habituation leading to increased resistance to TA and to common scab.

Among the seven TA-habituated somaclones regenerated in this study, three were tested for common scab resistance and one of them, the somaclone RB9, showed higher resistance to the disease than its non-habituated counterpart (Table 2). Additional *S*. *scabiei* infection trials in growth chambers revealed there was between 13 to 25% less infected tubers in RB9 than in RBP (Table 2). In addition to being more resistant to common scab, RB9 tubers were also less sensitive to TA as they showed less intense brown lesions than RBP tubers when treated with TA (Fig 2).

Field trials using RB9 and RBP plants grown in naturally infested fields at three different years (Tables 3 and 4) confirmed the improved resistance to common scab of somaclone RB9 compared to RBP. This enhanced resistance was characterized by a significant reduction in the CSSI of RB9 tubers compared to RBP tubers, except when the incidence of common scab was very low (Table 4, Trial 3). Moreover, the proportion of marketable tubers was increased by 7 to 22% in RB9 compared to RBP in field trials (Tables 3 and 4, Field trials 2 and 4). However, the average tuber weight and size of tubers grown in the field were significantly reduced in RB9 compared to RBP (Tables 4 and 5). It is possible that TA-habitation led to a reduction in tuber size in RB9. On the other hand, seed tubers used for field trials were generated in pots from *in vitro* grown plantlets. We can not exclude the possibility that plants grown in these conditions can produce heterogeneous tubers leading to uneven plant growth in the field. Additional field trials using homogenous seed tubers may be necessary to determine whether the reduction in RB9 tuber size was actually promoted by TA-habituation. Yet, the incidence of black scurf and internal defects such as hollow or brown heart and vascular discoloration as well as tuber specific gravity were not different in RB9 tubers compared to RBP tubers, suggesting that these physiological traits of the parental cultivar were conserved. Interestingly, it was shown that tubers from common scab resistant A380 mutant had increased resistance to powdery scab, soft rot and black scurf [32]. It would certainly be interesting to test whether the somaclone RB9 is also more resistant to these diseases.

The potato skin, or periderm, is the first barrier encountered by *S*. *scabiei* during tuber infection. Thangavel et al. (2016) have shown that *S*. *scabiei* infection leads to the thickening of the periderm’s phellem, a process enhanced in the common scab resistant A380 potato mutant that was obtained from somatic cell selection on high TA concentration [12, 31]. Interestingly, small RB9 tubers grown in the absence of *S*. *scabiei* already had a thicker phellem with an average of 13.3 cell layers compared to 11.5 in RBP (Fig 3A). Moreover, RB9 phellem cells showed a distorted shape compared to the mostly rectangular shape of RBP phellem cells (Fig 3B and 3C). Morphological changes in phellem cells may indicate altered suberin synthesis or composition in periderm cells, as observed in the A380 mutant [31]. While thickening of the phellem may contribute to prevent infection, further investigation will be required to determine whether changes in the RB9 phellem organization and suberin composition are also involved in protecting against *S*. *scabiei*. However, the observation of phenotypic changes induced both in the TA-habituated somaclone RB9 and the A380 mutant could reveal some of the key factors important for resistance to common scab.

Together, our results show that TA-habituation is a very efficient approach to improve resistance to common scab in a given cultivar. One out of the 3 TA-habituated somaclones initially tested in this study (Table 2) was more resistant to common scab than RBP, but it is possible that one or more of the 4 other TA-habituated somaclones was also resistant to common scab. We obtained similar results in another TA-habituation experiment performed in potato ‘Yukon Gold’ where 2 out of 7 TA-habituated somaclones tested for resistance to common scab showed a significant reduction in disease severity when compared to the parent cultivar [33]. Hence, this method could potentially be used to increase resistance to common scab in any potato cultivars, as long as it can produce calli and regenerate plantlets.

Our results also show that TA-habituation of potato calli not only reprogrammed cells to resist to TA but also led to the generation of a plant producing tubers with both reduced sensitivity to TA and increased resistance to common scab. Hence, the effects of TA-habituation were observed and maintained at the whole plant level in the regenerated plant. In addition to the occurrence of somatic genome variation between somatic cells [34], tissue culture techniques such as the production of calli and somatic embryo regeneration can generate somaclonal variations (genetic and/or epigenetic modifications associated with tissue culture) which could explain some of the changes observed in the somaclone RB9. Somaclonal variations may be stable over time and have been used to improve various traits in cultivated plants, including disease resistance [34–36]. On the other hand, based on the efficiency of the TA-habituation procedure in producing somaclones with enhanced resistance to common scab, we suggest that TA-habituation may in fact promote or select for genetic and/or epigenetic modifications that specifically lead to enhanced resistance to common scab through a yet to be understood mechanism. For instance, increased resistance to common scab could be due to enhanced or constitutive activation of defense- or stress-related pathways especially in tubers. Recent work using transcriptomic analysis that compared gene expression between common scab resistant and susceptible potato cultivars has revealed that immune priming may indeed be involved in the induced resistance in the common scab resistant cultivar Hindenburg [37]. However, there is wide evidence that the activation of defense mechanisms is a high energy demanding process that can lead to reduction in plant growth (rev. in [38, 39]). This type of defense could reduce tuber weight and size, as observed here for RB9 tubers. Interestingly, the TA-resistant Arabidopsis mutant *txr1* also showed some reduction in growth [13]. In this case, enhanced resistance to TA was associated with high ROS accumulation as well as enhanced plant immunity [24]. Whether TA-habituation in potato calli enhanced ROS production and/or plant immunity is currently under investigation.

## Conclusions

The TA-habituation method presented in this work was useful to enhance resistance to common scab in a widely cultivated potato cultivar. Further molecular and biochemical characterization of the RB9 somaclone will be invaluable to identify the changes that made this TA-habituated somaclone more resistant to common scab compared to its genetically equivalent cultivar Russet Burbank. This may ultimately lead to the identification of specific phenotypic characteristics (e.g., periderm thickness) or molecular markers that are linked with resistance to common scab.

## Supporting information

S1 Table **Tables A, B and C.** Common scab evaluation of individual tubers from original ’Russet Burbank’ (RBP) and TA-habituated somaclones cultivated in growth chamber (Trials 1, 2 and 3). **Table D.** Common scab evaluation for individual tubers from parent Russet Burbank (RBP) and TA-habituated somaclone RB9 (Field evaluation 2012). **Tables E, F and G.** Common scab evaluation of individual tubers from original ’Russet Burbank’ (RBP) and TA-habituated somaclone RB9 cultivated in the field (Field trials 2, 3 and 4). **Tables H, I, J and K**. Weight of individual tubers from original ’Russet Burbank’ (RBP) and TA-habituated somaclone RB9 cultivated in the field (Field trials 1 to 4). **Table L.** Yield of tubers as a function of tuber size for parent cultivar Russet Burbank (RBP) and somaclone RB9 grown in the field. **Table M.** Evaluation of physiological characteristics of tubers from Russet Burbank parent (RBP) and somaclone RB9 grown in the field.
(XLSX)
